# In Vivo *HOXB7* Gene Silencing and Cotreatment with Tamoxifen for Luminal A Breast Cancer Therapy

**DOI:** 10.3390/ph17101325

**Published:** 2024-10-04

**Authors:** Ana Beatriz Caribé dos Santos Valle, Fábio Fernando Alves da Silva, Maria Ângela Pepe Carneiro, Bruno Espuche, Guilherme Diniz Tavares, Emerson Soares Bernardes, Sergio Enrique Moya, Frederico Pittella

**Affiliations:** 1 Laboratório de Desenvolvimento de Sistemas Nanoestruturados, Faculdade de Farmácia, Universidade Federal de Juiz de Fora, Rua José Lourenço Kelmer, Juiz de Fora 36036-900, Brazil; abcsvalle@hotmail.com (A.B.C.d.S.V.); guilherme.tavares@ufjf.br (G.D.T.); 2 Instituto de Pesquisas Energéticas e Nucleares, Centro de Radiofarmácia (IPEN/CECRF), Comissão Nacional de Energia Nuclear, São Paulo 05508-000, Brazil; fabiofufg@gmail.com (F.F.A.d.S.); mangelapc@usp.br (M.Â.P.C.); emerson.bernardes@gmail.com (E.S.B.); 3 Soft Matter Laboratory, Center for Cooperative Research in Biomaterials (CIC biomaGUNE), Basque Research and Technology Alliance (BRTA), Paseo Miramón 194, 20014 Donostia-San Sebastián, Spain; bruno.espuche@gmail.com (B.E.); smoya@cicbiomagune.es (S.E.M.)

**Keywords:** breast cancer, tamoxifen, hybrid nanoparticles, RNA interference, siRNA

## Abstract

Background: Acquired resistance and adverse effects are some of the challenges faced by thousands of Luminal A breast cancer patients under tamoxifen (TMX) treatment. Some authors associate the overexpression of HOXB7 with TMX resistance in this molecular subtype, and the knockdown of this gene could be an effective strategy to regain TMX sensitivity. Therefore, we used calcium phosphate hybrid nanoparticles (HNP) for the delivery of short interfering RNA molecule (siRNA) complementary to the HOXB7 gene and evaluated the RNA interference (RNAi) effects associated with TMX treatment in breast cancer in vivo. Methods: HNP were prepared by the self-assembly of a methoxy-poly (ethylene glycol)-block-poly (L-glutamic acid) copolymer (PEG-pGlu) and the coprecipitation of CaPO_4_ to incorporate siRNA. The in vitro cell viability and migration were evaluated prior to in vivo experiments. Further, animals bearing early-stage and advanced Luminal A breast cancer were treated with HNP-siHOXB7, HNP-siHOXB7 + TMX, and TMX. Antitumoral activity and gene expression were evaluated following histopathological, hematological, and biochemical analysis. Results: The HNP were efficient in delivering the siRNA in vitro and in vivo, whilst HOXB7 silencing associated with TMX administration promoted controlled tumor growth, as well as a higher survival rate and reduction in immuno- and hepatotoxicity. Conclusions: Therefore, our findings suggest that HOXB7 can be an interesting molecular target for Luminal A breast cancer, especially associated with hormone therapy, aiming for adverse effect mitigation and higher therapeutic efficacy.

## 1. Introduction

Breast cancer is currently the most common type of cancer affecting women worldwide. Even more concerning, the mortality rate of this disease grows every year [1]. Traditional treatments of cancer, such as surgery, radio-, and chemotherapy, were introduced prior to the 1970s and still play a major role in the cancer approach. However, hormone therapies that pharmacologically alter oncogenic pathways have advanced in recent years. Furthermore, it was only at the turn of the century that FDA-approved specific and targeted therapies, such as the use of monoclonal antibodies and antibody-drug conjugates, gained relevance [2]. Currently, several strategies are being studied, but specific genetic alterations can be achieved via gene editing and RNA interference therapy.

Among breast cancer molecular subtypes, Luminal breast cancer (positive hormone receptor molecular subtype) is responsible for approximately 70% of all breast cancer cases, which justifies the need for novel therapeutic approaches against this tumor type [3]. In most cases, hormone therapy is the recommended treatment, and the first-line medicine used for this molecular subtype is TMX, a selective estrogen receptor, that, in breast tissue, acts as an antagonist that blocks the proliferation signaling pathway activated by this receptor [3]. However, acquired resistance is frequent, and 40% of recurrences do not respond to endocrine therapy [4]. Among some molecular mechanisms that lead to endocrine resistance, the enhanced activity of EGFR/HER2, ER, and MAPK pathways by HOXB7 overexpression has been highlighted [4]. A high expression of the HOXB7 gene is associated with tumorigeneses, angiogenesis, cell invasion, proliferation, and survival, as well as the evasion of TMX blockage [5,6,7,8,9]. In addition to TMX resistance, adverse effects caused by this therapeutic agent also raise immunological, health, and aesthetic concerns.

The first RNAi-based medicines have been approved by regulatory organs in 2018 [10]. Medicines based on the RNAi effect comprise a post-transcriptional gene expression regulation and consist of the delivery of a small double-stranded RNA, which acts by cleaving a target messenger RNA (mRNA) with the help of an intracellular ribonucleoprotein complex. To achieve the sequence-specific effect in a targeted organ, synthetic molecules known as siRNA must surpass several biological barriers to enter the cell cytoplasm [11]. Furthermore, direct administration of the siRNA in the bloodstream leads to rapid clearance, unspecific interactions, and enzymatic degradation. Therefore, to ensure siRNA delivery and RNAi effect, the use of nanoparticles as carriers has been an effective strategy to protect the nucleic acid and assure not only cellular uptake but also endosomal escape and cytoplasmic release [12].

The application of nanotechnology in the biomedical and pharmaceutical fields is explored in the construction of smart nanocarriers. Currently, many studies demonstrate the use of nanoparticles of different materials as drug delivery systems. Nanoparticles made only with organic materials (such as polymeric micelles, liposomes, solid lipid nanoparticles, and chitosan nanoparticles), with inorganic materials (such as iron oxide nanoparticles and gold nanoparticles), or with a mixture of both materials (hybrid nanoparticles), have been studied as drug delivery systems for breast cancer treatment [13,14,15,16,17,18]. Similarly, several nanoparticles show improved characteristics to be used to deliver siRNA [18,19].

Among a diversity of delivery systems, hybrid nanoparticles composed of an inorganic calcium phosphate core covered by PEG-block-polyanionic copolymers are a promising strategy for delivering genetic material. In animal models, these nanocarriers could avoid severe immune response, unspecific macromolecule interactions, nuclease degradation, and off-target accumulation while promoting passive endothelial penetration and endosomal escape with negligible toxicity [20,21,22,23]. Thus, this work aimed to apply these hybrid nanoparticles to deliver siRNA molecules to human breast cancer tumors in nude mice and assess the effects of HOXB7 gene silencing with and without TMX cotreatment in vivo.

## 2. Results

### 2.1. Hybrid Nanoparticle Preparation, Characterization, and Purification

**Figure 1:**
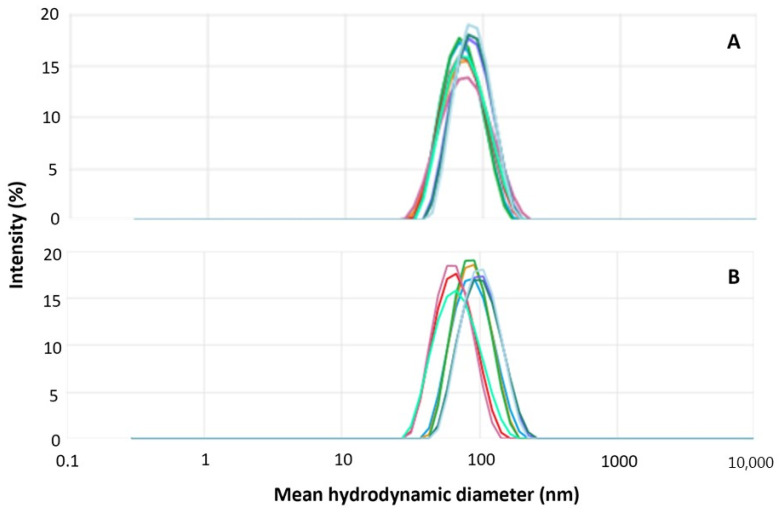
Size distribution curve of HNP-siHOXB7 (**A**) and HNP-mock (**B**) by intensity. Each curve in a different color represents one single measurement (n = 9).

Hybrid nanoparticles were prepared via the self-assembly of PEG-pGlu in the presence of Ca^+2^ and phosphate ions. Nanoparticles containing HOXB7 siRNA (HNP-siHOXB7) and without siRNA molecules (HNP-mock) were prepared and further characterized. Macroscopically, a translucent and precipitate-free dispersion was obtained. Figure 1 shows the size distribution by intensity of HNP-siHOXB7 and HNP-mock. The mean hydrodynamic diameter, polydispersity index (PDI), and Zeta potential are presented in Table 1.

DLS technique revealed a unimodal size distribution (Figure 1) that, in combination with a PDI of 0.1 for both samples, indicates a unimodal distribution. HNP-siHOXB7 and HNP-mock presented mean hydrodynamic diameters of 74.4 and 82.2 nm, respectively, and a Zeta potential close to zero. Furthermore, to eliminate excess calcium ions for in vivo application, HNP were submitted to a purification process and were further characterized. As observed in Table 2, the original properties of both HNP were not altered, and nanoparticles were suited for systemic applications even 6 days after purification.

**Figure 2:**
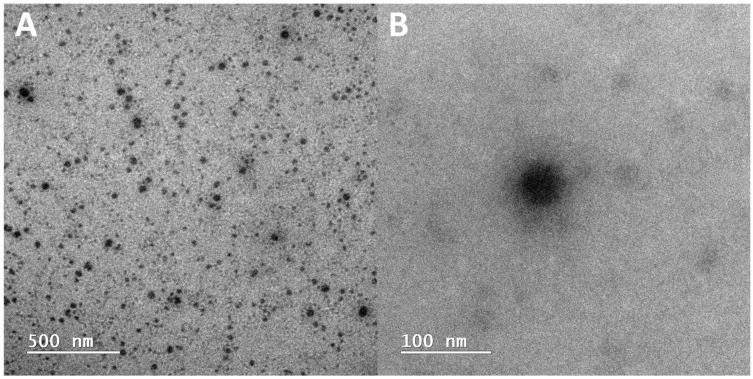
Representative images of hybrid nanoparticles by TEM. (**A**) Scale bar = 500 nm and (**B**) scale bar = 100 nm.

Transmission electronic microscopy (TEM) resulted in a similar outcome compared to other studies [21,22,23,24]. A homogeneous and spherical morphology for the nanoparticles was observed (Figure 2). Furthermore, siRNA encapsulation efficiency (EE) by the HNP containing 1000 µg/mL of PEG-pGlu was 52.5 ± 2.9%.

Also, the colloidal stability of HNP stored at 4 °C was evaluated for 180 days (Supplementary Figure S1). At the end of the experiment, no significant differences (*p* > 0.05) were observed regarding HNP-siHOXB7 size and PDI comparing days 1 and 180 (Supplementary Table S1). HNP-mock particles remained stable until day 80; however, at the end of the experiment, the particle size showed a slight increase (*p* < 0.05).

**Figure 3:**
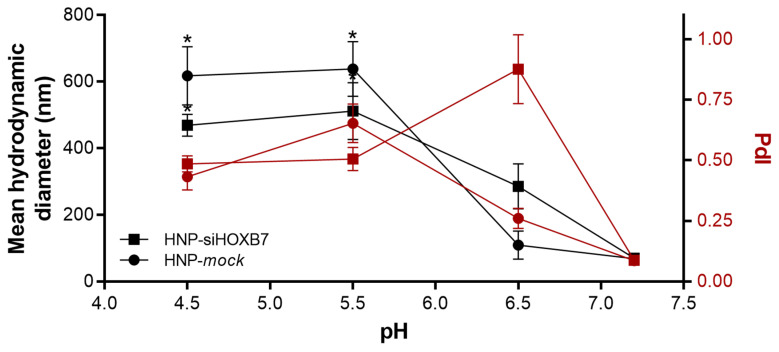
Stability of HNP-siHOXB7 (square, n = 3) and HNP-mock (circle, n = 3) after exposure to acid pH. Mean hydrodynamic diameter (black) and PDI (dark red). Results are expressed as mean ± standard error of the mean. ANOVA followed by Bonferroni (* *p* < 0.05).

The cell entry route of most nanoparticles is the endocytic pathway [19,25]. Some authors have already confirmed that similar HNP were internalized by endocytosis and were able to promote endosomal escape and cytoplasmic siRNA release [21]. Here, HNP were submitted to pH conditions to mimic the endosomal environment. Figure 3 shows that HNP dispersion is stable at physiological pH of approximately 7.2; however, in acidic conditions, a high destabilization and size increase, most likely due to HNP aggregation, is observed (*p* < 0.05).

Finally, freeze-drying stability was also assessed aiming for a longer storage period and future transportation of the formulations. To avoid HNP aggregation and the loss of its original properties during this process, sucrose 10% was added to the dispersion as a cryoprotector. After freeze drying, HNP were resuspended in Milli-Q water and reanalyzed (Table 3). Both samples remained stable after resuspension, and no differences in size and PDI were detected (*p* > 0.05). However, after 10 days of resuspension, the cryoprotectant-added sample doubled in size and tripled PDI values (*p* < 0.05).

### 2.2. Cell Viability

**Figure 4:**
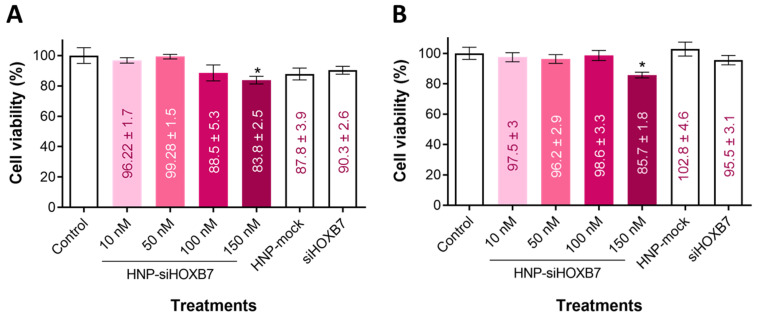
Cell viability of MCF7 cells (n = 6) by MTT assay after (**A**) 24 h and (**B**) 48 h. Results are expressed as mean ± standard error of the mean (n = 6). ANOVA followed by Bonferroni (* *p* < 0.05).

Cell viability was assessed in the Luminal A molecular subtype MCF7 cell line using the MTT assay (Figure 4). After 24 and 48 h, a slight reduction in cell viability in the highest concentration tested (*p* < 0.05) was observed. Most importantly, empty HNP and free siHOXB7 did not reduce viability in both times tested (*p* > 0.05).

### 2.3. Cell Migration

**Figure 5:**
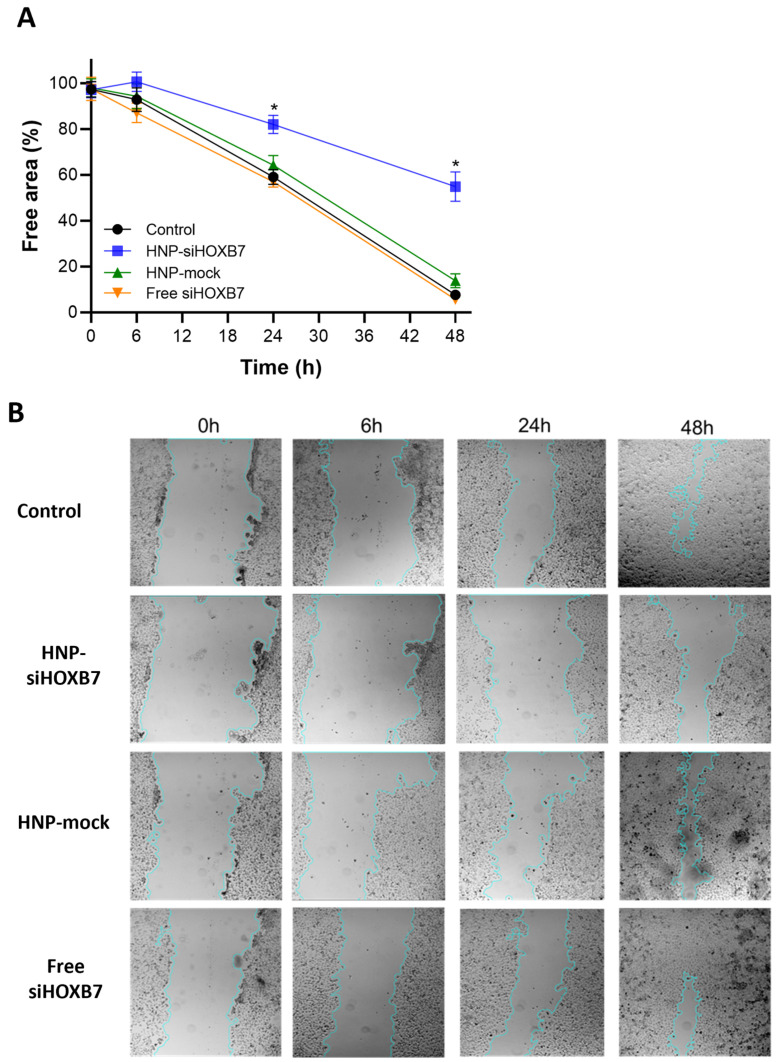
Migration of MCF7 cells after 6 h, 24 h, and 48 h of treatment (n = 3). (**A**) Graphic showing the percentage of free area versus time of treatment and (**B**) Representative images of the scratch and each treatment, showing the evolution of the same scratched area over time. Results are expressed as mean ± standard error of the mean. ANOVA followed by Bonferroni (* *p* < 0.05).

Since the HOXB7 gene is also related to migratory pathways, cancer cell migration was assessed after treatment with HNP-siHOXB7 by the scratch assay in the MCF7 cell line. A significant difference in the free area was observed on HNP-siHOXB7-treated cells (*p* < 0.05) 24 h after the treatment. Additionally, another expressive inhibition of migration was observed 48 h after HNP-siHOXB7 treatment. On the contrary, HNP-mock and free siHOXB7 groups presented scratch closure similar to the control group. After 48 h, the HNP-siHOXB7 treated group presented a free area of 52%, whereas control groups fluctuated between 5 and 13% (Figure 5).

### 2.4. Hemolysis Assay

**Figure 6:**
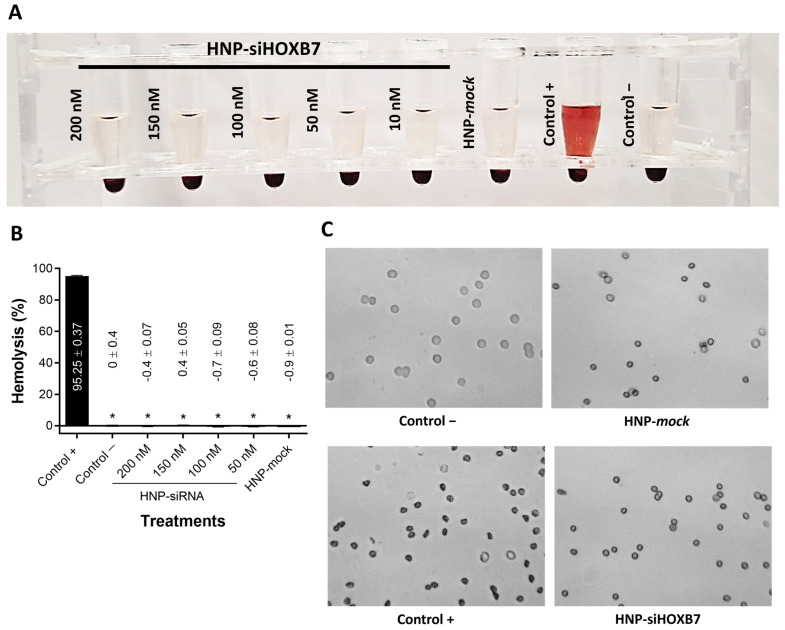
Hemolytic activity of HNP formulations (n = 3). (**A**) Macroscopic observation of hemolysis, (**B**) Graphic showing the percentage of hemolysis in each group of treatment, and (**C**) Representative microscopic images of red blood cells after each treatment. Results are expressed as mean ± standard error of the mean. ANOVA followed by Bonferroni (* *p* < 0.05).

Before animal experimentation, a hemolysis test was performed to evaluate any possible toxicity to red blood cells by the HNP. Thus, different HNP concentrations were evaluated visually by spectrophotometer and by optic microscopy (Figure 6). There was no hemolytic activity promoted by the HNP-siHOXB7 or HNP-mock (*p* > 0.05) in any of the tested concentrations, which indicates non-toxicity by the components of the nanoparticles (Figure 6A,B). In accordance, positive control promoted hemolysis of the red blood cells (*p* < 0.05) as observed both visually and by spectrophotometer (95%). On the contrary, negative control, HNP-siRNA, and HNP-mock treatments presented no hemolysis (0%). As observed in Figure 6C, these cells presented irregular membranes and smaller sizes compared to negative control and other treatments.

### 2.5. Antitumoral Activity and Gene Expression Evaluation

**Figure 7:**
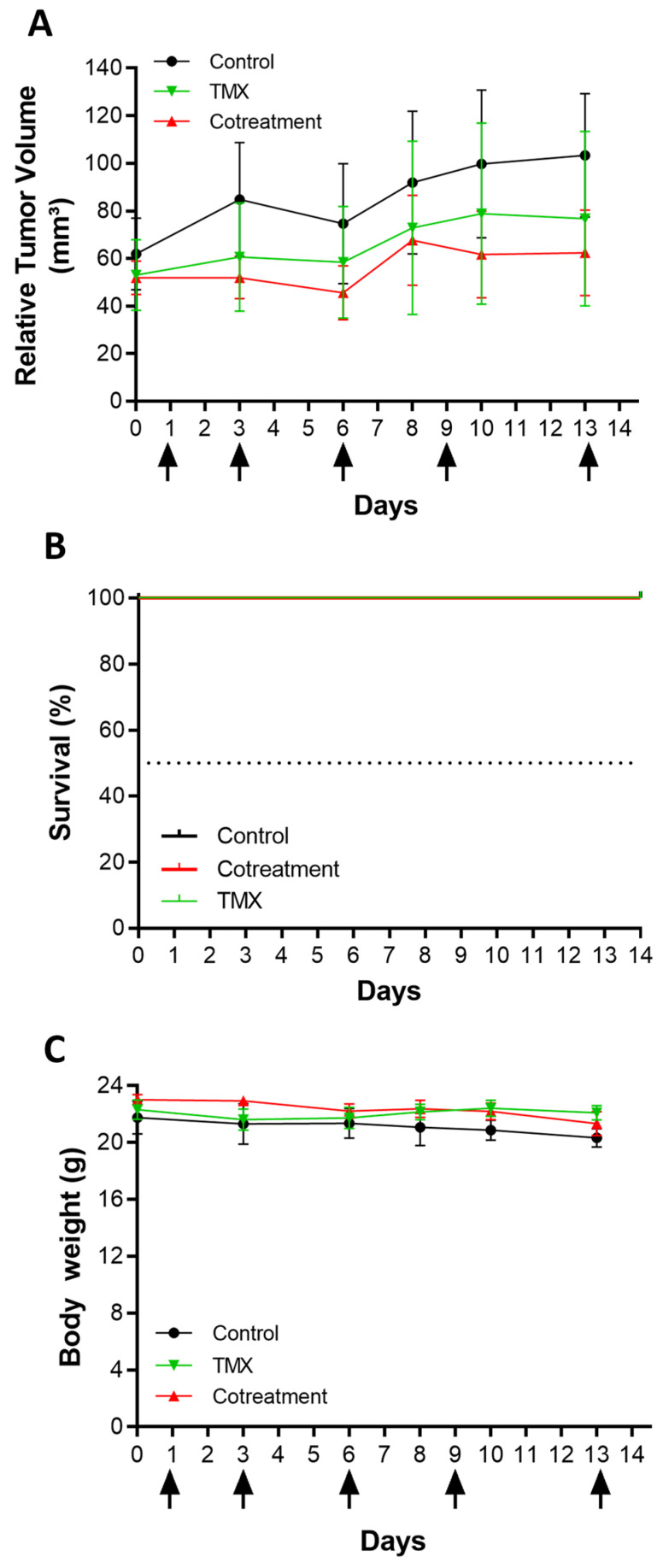
Antitumoral activity in nude mice bearing early-stage tumors (50 mm^3^) was monitored for 14 days. (**A**) Relative tumor volume, (**B**) survival rate, and (**C**) body weight variation. The arrows indicate days of injection. Black, green, and red lines represent control (n = 4), TMX (n = 4), and cotreatment (n = 4) groups. Dotted line is 50% of the survival rate. Results are expressed as mean ± standard error of the mean (SEM).

**Figure 8:**
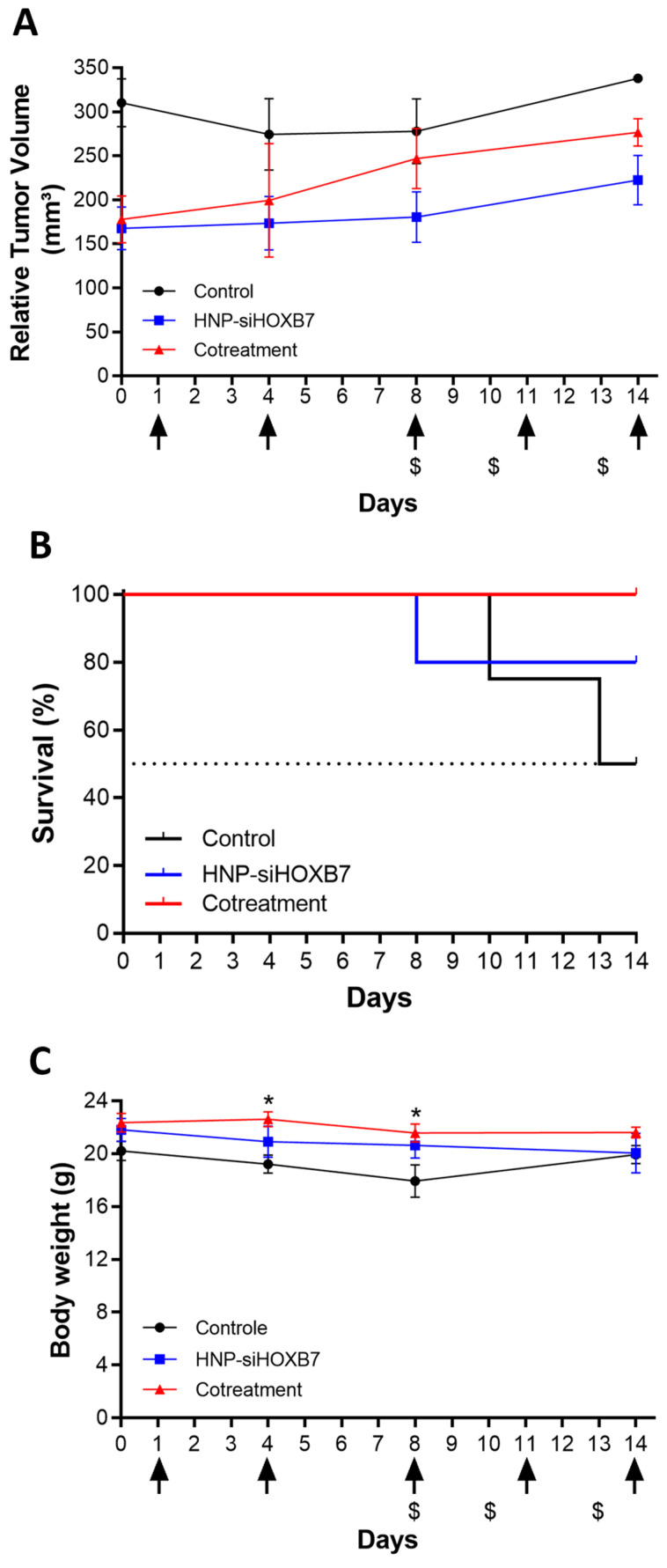
Antitumoral activity in nude mice bearing advanced tumors (250 mm^3^) was monitored for 14 days. (**A**) Relative tumor volume, (**B**) survival rate, and (**C**) body weight variation. The arrows indicate days of injection, and $ symbols indicate days of death. Black, blue, and red lines represent control (n = 4), cotreatment (n = 4), and free TMX (n = 4) groups. Results are expressed as mean ± standard error of the mean (SEM). ANOVA followed by Bonferroni (* *p* < 0.05).

To evaluate the impact of HOXB7 gene silencing on tumor growth, nude mice bearing early-stage (50 mm^3^), and advanced tumors (250 mm^3^) were intravenously injected with HNP-siHOXB7. The cotreatment of mice with HNP-siHOXB7 and TMX led to controlled tumor growth after 13/14 days compared to control groups (Figure 7 and Figure 8) in both scenarios. Importantly, in the advanced tumors assay, two animals were found dead during the assay, leading to a distinct survival rate over the treatment. Thus, HNP-siHOXB7 and cotreatment groups improved the survival rates to 80 and 100%, respectively, in the period, while the control group (no treatment) presented a reduced survival rate of 50% in advanced tumors (Figure 8B). Furthermore, no significant body weight loss was detected at the end of the experiment (Figure 7C or Figure 8C).

**Figure 9:**
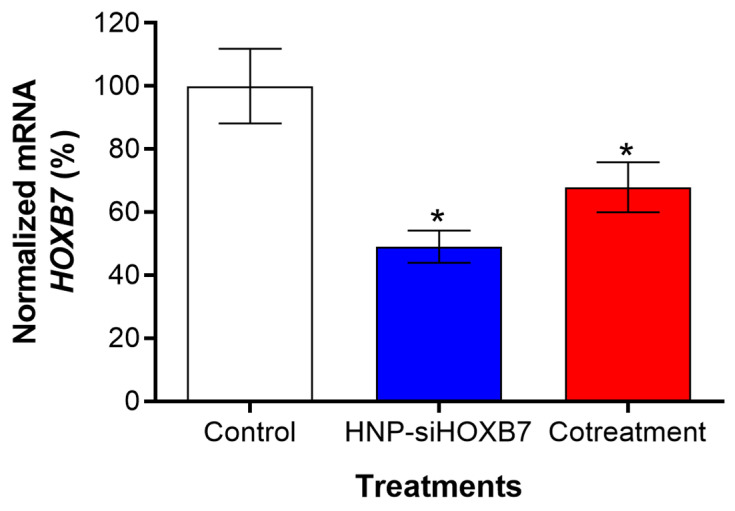
HOXB7 gene expression in breast tumor tissue after HNP-siHOXB7 and cotreatment. Controls were set at 100% of HOXB7 expression and normalized by the β-actin house-keeper gene. Results are expressed as mean ± standard error of the mean (SEM). ANOVA followed by Bonferroni (* *p* < 0.05).

To further evaluate the behavior of advanced tumors and to understand whether the controlled tumor growth was related to HOXB7 gene silencing by RNAi effect from HNP-siHOXB7 treatment, real-time qPCR was performed in tumor tissue. Interestingly, as shown in Figure 9, the expression of HOXB7 was reduced by 50% and 32% after HNP-siHOXB7 and cotreatment, respectively (*p* < 0.05).

### 2.6. Histopathological Evaluation

Histopathological analysis of lung, spleen, heart, liver, kidney, and tumors was carried out to evaluate tissue damage or inflammation due to treatment exposure. Early-stage tumors (50 mm^3^) groups presented similar histopathological characteristics between control and treated groups. However, the TMX group (n = 4) showed interstitial chronic spleen (75%), kidney (75%) congestion, and neutrophilic infiltration (25%).

**Figure 10:**
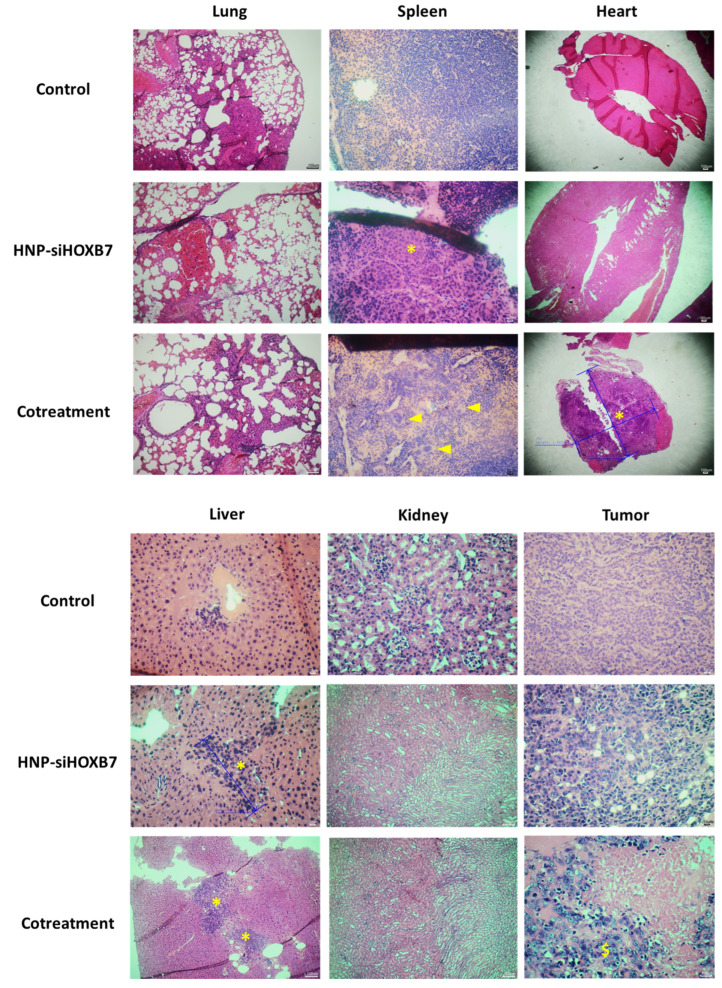
Representative images of histopathology analysis of organs and tumors after treatment with HNP-siHOXB7, cotreatment in advanced tumors. Micro- and metastasis were found in the spleen and liver in the HNP-siHOBXB7 group and the heart and liver in the cotreatment group (asterisk). Cotreatment group presented high splenic extramedullary hematopoiesis (arrows) and tumor necrosis ($ symbol). Tissues (n = 4/5) were stained with H & E, and images were acquired at a scale of 100 (heart) and 10 µm.

Individuals with advanced tumors (250 mm^3^), however, exhibited more severe histopathological characteristics, as expected (Figure 10). The two control animals left displayed extramedullary erythropoiesis in the spleen and discrete hepatic congestion in the liver and kidney, which was similar to HNP-siHOXB7 and the cotreatment group. One animal in HNP-siHOXB7 presented micrometastasis in the liver and spleen (n = 1), while the other 4 did not present such characteristics. Animals in the cotreatment group presented lymphoid hyperplasia (n = 4). Micrometastasis in the heart and liver was found in one animal (n = 1) of the cotreatment group, while the other three did not present such characteristics. Necrosis was found in the tumor tissue of three out of the four animals (75%) of the cotreatment group, as well as the angiolymphatic invasion of tumor cells in one animal (25%).

### 2.7. Hematological and Biochemical Parameters Analysis

The hematological and biochemical parameters were determined to understand the in vivo toxicity of both cotreatment and free TMX in early-stage tumors (Table 4). Overall, a significant decrease in white blood cells and plasma proteins was observed in the free TMX group (*p* < 0.05). In fact, the only relevant difference regarding the cotreatment group was the increase in creatinine (*p* < 0.05). Most importantly, however, the value of aspartate aminotransferase (AST) and alanine aminotransferase (ALT) levels in the HNP-siHOXB7 treated group remained in standard amounts similar to non-treated animals.

## 3. Discussion

The HOXB7 gene is known to be related to TMX resistance in positive hormone receptor molecular subtype breast cancer. Therefore, therapeutic strategies involving HOXB7 silencing in combination with TMX administration may be a promising future approach in the treatment of TMX-resistant cells from this molecular subtype. The combination therapy was proposed in this study, and HOXB7 silencing associated with TMX showed controlled tumor growth and reduction in immuno- and hepatotoxicity. Furthermore, this strategy resulted in a higher survival rate if compared to the control and HNP-siHOXB7 groups.

To achieve this, hybrid nanoparticles composed of calcium phosphate and a block copolymer containing PEG-pGlu were produced as in previous reports [22,23]. The size distribution, mean hydrodynamic diameter, PDI, and Zeta potential were in accordance with other authors [20,21,22,23,24], which shows a high reproducibility of the preparation of these nanoparticles that consists of a rapid and simple mixture of constituents in stoichiometric conditions. It is known that calcium phosphate crystals grow uncontrollably during wet precipitation. Thus, we controlled the particle size below 100 nm by using PEG-pGlu (Figure 1, Table 1). Sizes below 100 nm are desired in intravenous cancer therapy, especially if the targeting is dependent on the enhanced permeability and retention effect (EPR), which is the case [25,26]. The neutral surface charge (Table 1) provided by the non-ionic PEG portion can be advantageous since it creates a hydrophilic layer that promotes lower immunogenicity, which leads to higher circulation time and bioavailability and enhanced probability of tumor accumulation. Furthermore, TEM images (Figure 2) confirmed HNP size, and the monodisperse character pointed by a unimodal size distribution curve (Figure 1) and 0.1 PDI values (Table 1). Also, rounded nanoparticles observed by TEM do not present orientational dependence; therefore, blood dislocation and cell incorporation may be facilitated [25]. Thus, HNP presented desirable characteristics for intravenous applications for anticancer treatment [26,27,28,29].

An incorporation ratio of siRNA into HNP of 50% was observed, which is comparable to other works published using similar nanoparticles. In this formulation, the siRNA incorporation ratio is competitively controlled by the copolymer concentration, which in turn also affects colloidal stability. Therefore, the initial concentration of 1000 µL/mL of PEG-pGlu was chosen to carry out in vivo experiments.

The colloidal stability of non-purified HNP stored at 4 °C was assessed for 180 days and revealed no difference in the tested parameters (Figure S1, Table S1), which indicates long-term storage potential. Purified HNP (Table 2) did not show a significant difference in size and PDI for at least 6 days, which indicates high colloidal stability of the complex for immediate systemic administration. A purification process is necessary in this type of nanocarrier to remove the excess of free calcium ions, which can lead to changes in cardiovascular homeostasis [30]. An established methodology was performed to this end based on Souza et al., who reported an 88% and 56% removal of residual calcium and phosphorous, respectively [23]. In any case, the lyophilization process was assessed since it allows even higher stability of the purified formulation. Even though cryoprotectants are usually used for nanoparticle protection and aggregation avoidance, sucrose 10% addition did not fulfill this requirement and reduced the colloidal stability of HNP after 10 days of resuspension (Table 3). Thus, no cryoprotectants were needed in this process since the PEG layer was able to protect the HNP from the stress of the process and acted as a cryopreservant.

Nanoparticle disassembly was assessed by mimicking the endocytic environment by pH variation. After exposing the HNP to acidic conditions, the HNP underwent rapid disorganization/dissolution (Figure 3). According to our previous work [14], this dissolution leads to an ion gradient increase followed by endosome membrane rupture due to water influx and siRNA release to the cytoplasm. Other authors observed a similar effect after lower pH exposure of HNP [31]; however, the optimization of the nanoparticles can be further assessed to avoid early release of siRNA once the destabilization of the system started at 6.5 pH, and this pH is commonly found in tumoral microenvironment [32].

Other than reduced viability by HNP-siHOXB7 treatment, empty HNP (HNP-mock) presented no cytotoxicity, which confirms the safety of the formulation. No toxicity was observed in cells treated with free siHOXB7, which indicates that this molecule is not capable of entering cells or escaping from the endosomal compartment and therefore requires a delivery system to this end (Figure 4). Regarding the scratch assay, the group treated with HNP-siHOXB7 significantly reduced the scratched area, indicating a reduced migration of cells after HNP-siHOXB7 treatment (Figure 5). This suggests that HOXB7 silencing may have an anti-migratory potential in MCF7 cells and may play an important role in reducing tumoral progression and metastasis. Similarly to the cell viability assay, the HNP-mock and free siHOXB7 presented the same wound area closure pattern as the control group in the scratch assay. Taken together these results suggest siRNA internalization by the cells following the RNAi effect, since the HOXB7 gene is associated with cell proliferation and migration. In fact, a previous work of our group showed that HNP were able to deliver siRNA to breast cancer cells for a significant HOXB7 gene silence in vitro [33].

The development of efficient and non-toxic nanocarriers is still the main challenge in drug and gene delivery in animal experiments [34]. To confirm the ability of the HNP to deliver siRNA molecules in a mouse model, HNP were first submitted to a hemolysis assay to ensure HNP non-toxicity to red blood cells (Figure 6). No hemolytic activity was found, and cells presented normal morphology after HNP exposure. On the contrary, cells from the positive control group presented a morphological anomaly called echinocyte. This results from extravasation of intracellular content by changes in membrane fluidity and lipidic redistribution, leading to smaller red blood cells [35].

Antitumoral activity was then assessed in early-stage (50 mm^3^, Figure 7) and advanced (250 mm^3^, Figure 8) Luminal A tumors. HOXB7 gene is found to be overexpressed in the MCF7 cell line [7], and it is involved in several metabolic pathways of cancer. In breast cancer, this gene was already associated with tumorigeneses, angiogenesis, DNA repair, cell invasion, proliferation and survival, and drug resistance, especially related to TMX [5,6,7,8]. Since HOXB7 plays a role in different areas of breast cancer progression, Jin et al. [5] proposed that this gene could be an interesting molecular target for antitumoral therapy. In fact, they showed TMX resistance association to HOXB7 overexpression in TMX-resistant MCF7 cells, and HOXB7 abrogation led to TMX sensitization through EGFR/HER2 and ER pathways, which are commonly associated with drug resistance. In the present study, a controlled tumor volume growth was observed after HOXB7 knockdown by HNP-siHOXB7 associated or not with TMX (Figure 7A or Figure 8A). Although the overall tumor volume was not reduced, an expressive tumor regression should be associated with a longer period of experimentation in observation of the low proliferation rate of Luminal A cell lines, as noted by Jin et al. [5]. Nonetheless, an enhanced survival rate in treated advanced tumor groups was also observed here (Figure 8B). This was detected, especially in combination with TMX, and the combination therapy strategy presented superior effects if compared to HOXB7 knockdown alone.

In fact, by reducing HOXB7 gene expression, we expect to re-sensitize Luminal A breast cancer to TMX. The possible synergic effect of HOXB7 knockdown and TMX was previously assessed in vitro in MCF7 cells and demonstrated to have a potential antitumoral effect over HOXB7 depletion alone [33]. It is also important to mention that the mechanisms of TMX resistance are not exclusively to HOXB7 gene overexpression, and metabolic reprogramming in glucose, lipid, and amino acid metabolisms, as well as nucleic acid metabolism and mitochondria accumulation of TMX, can implicate in its resistance [13]. Noteworthy, these results were corroborated in this work by a significant HOXB7 knockdown in vivo after real-time PCR analysis (Figure 9). Importantly, the death of animals compromised the detection of statistical significance (Figure 8A) and indicates the need for increasing animal numbers. Moreover, aiming for an enhanced antitumoral effect, siRNA concentration can be safely increased to 200 nM without presenting any hemolytic toxicity, as previously shown (Figure 6).

Also, animals did not present signs of acute toxicity due to treatments once no weight loss or stress-related behavioral changes were observed (Figure 7C or Figure 8C). Most of the histopathological observations presented alterations caused by the neoplasia and TMX adverse effects (Figure 10). This suggests that HNP treatment did not promote any tissue toxicity and confirms the non-toxic character of the formulation. Although micro-metastasis was spotted in 1 animal (20%) of siHOXB7 and cotreatment groups, the other animals of those groups (80%) did not present such observations. In fact, the evaluation of metastatic regions is of great importance and should be properly investigated in future experiments with larger samples as a possible cause of death in the control group. In accordance, the reduced number of animals with micro-metastasis in HNP-siHOXB7 and cotreatment groups (20%) could be due to reduced cancer cell migration as HOXB7 depletion reduced migratory potential of MCF7 cells in vitro (Figure 5). In addition, hematological and biochemical parameters analysis resulted in less overall toxicity of cotreatment (Table 4). Noteworthy, neither ALT nor AST enzyme levels were increased after HNP administration. This shows an absence of hepatocellular toxicity by intravenously injected HNP, and it is highly desired for formulations containing nanoparticles since this is one of the reasons for nanoparticle-related clinical trial failure [34]. Therefore, our results support that HOXB7 silencing, especially if associated with TMX, promoted a better prognosis, extended general survival, and reduced overall toxicity.

Clinical translation is a major challenge in RNAi-based nanomedicines, and frequently, in vitro results are not reproduced after in vivo administration of these formulations. A successful transfection includes overcoming a variety of biological barriers to promote the RNAi effect in the desired tissue. Therefore, the delivery system not only protects the siRNA molecule from degradation enzymes and reticuloendothelial system but also must be able to overflow the tumoral tissue, resist extracellular medium, transpose cellular membrane, promote endosomal escape, and, finally, release the siRNA molecules into the cytoplasm where they can promote the RNAi effect [19]. Hence, the importance of the present work and potential clinical translation of the formulation, since it was observed a successful siRNA transfection and, most importantly, the absence of general toxicity.

New formulations must ensure safety, specificity, and off-target effect avoidance. Single-cell sequencing analysis and bioinformatics can assist in finding genes and/or proteins that can be not only potential therapeutic targets but also have an important role in active targeting [36]. Also, convertible charge polymers can assist in the release of the material in environments with a particular characteristic [37]. Furthermore, this research also acknowledges the combinatorial strategy of RNAi therapy and hormone therapy and can be a step forward in establishing or remodeling therapeutic protocols.

## 4. Materials and Methods

### 4.1. Materials, Cell Line, and Animals

Primers and siRNA were purchased from Sigma-Aldrich^®^ (São Paulo, Brazil). Primer sequences are (5′-3′): CCAACCGCGAGAAGATGA (β-actina forward), CCAGAGGCGTACAGGGATA (β-actina reverse), ACCGACACTAAAACGTCCCT (HOXB7 forward) e AAACCGAACTTGAGGCTGGA (HOXB7 reverse). The siHOXB7 sequences are 5′ ACCUACCACUCGCGUGUUC dTdT 3′ (sense) and 5′ GAACACGCGAGUGGUAGGU dTdT 3′ (antisense). Methoxy-poly (ethylene glycol) -block-poly (L-glutamic acid) copolymer (PEG-pGlu) (sodium salt) was purchased from Alamanda Polymers, Inc. (Huntsville, AL, USA). The Amicon ultrafilters were purchased from Millipore (Burlington, MA, USA). Pi-Clear RNA Total was acquired from Pi-Biotech Genética Avançada Ltd.a (Juiz de Fora, Brazil). High-capacity cDNA Reverse Transcription kit was purchased from Applied Biosystems (Foster City, CA, USA). qPCR-SYBR-Green mix was purchased from Ludwig Biotecnologia Ltd.a (Rio Grande do Sul, Brazil). The cell line used for in vitro and in vivo assays was MCF-7, a Luminal A subtype breast cancer cell line.

Female nude mice (6–8 weeks old) were housed in individual cages covered with sawdust, which was replaced weekly. The animals were kept at a controlled temperature (23 ± 1 °C), 12 h light/dark cycle, and food and water ad libitum. The animals were constantly evaluated regarding pain, stress, and discomfort to evaluate the necessity of a humanitarian endpoint. All animal work was performed with the approval of the Ethics Committee for Animal Use from Instituto de Pesquisas Energéticas e Nucleares, Brazil (49/23).

### 4.2. Preparation of Hybrid Nanoparticles

Hybrid nanoparticles (HNP) were prepared by simple mixing, allowing self-assembly of the components as described by Pittella et al. [21] with modifications. Briefly, PEG-pGlu was diluted in TRIS-HCl buffer solution (10 mM, pH 7.4) to a concentration of 1000 µg/mL. The copolymer solution was added to a HEPES buffer (50 mM) containing Na_2_PO_4_ (1.5 mM) and NaCl (140 mM) and a 15 µM siRNA solution (2.5:7.5:2.5; *v*/*v*). Another solution was prepared by adding 2.5 M CaCl_2_ (solution to 10 mM TRIS buffer at pH 10 (1:11.5; *v*/*v*). The nanoparticle dispersion was obtained by pipette mixing both solutions to a final siRNA concentration of 1.5 µM. Hybrid nanoparticles containing siHOXB7 were named HNP-siHOXB7. Before animal administration, the HNP were purified using Amicon centrifugal filters (MW cut-off 30 kDa) at 15,000 rpm for 20 min at 4 °C.

### 4.3. Physicochemical Characterization of Hybrid Nanoparticles

Mean hydrodynamic diameter, polydispersity index (PDI), and size distribution of HNP were analyzed by Dynamic Light Scattering (DLS) measurements using Zetasizer Ultra equipment (Malvern Instruments, Malvern, UK) with a detection angle of 173° with He-Ne laser (633 nm) as the incident beam. The Zeta potential was evaluated by the same equipment by the electrophoretic mobility technique. Three independent NP were analyzed at 25 °C for each physicochemical characterization and colloidal stability study.

The morphology of the dispersion was observed through transmission electron microscopy (TEM) using JEOL JEM-1400 PLUS equipment (Jeol Ltd., Tokyo, Japan) at a voltage acceleration of 40–120 kV and operated in bright field mode. HNP-siHOXB7 was applied on carbon grids and dried for 10 min at room temperature.

### 4.4. Colloidal Stability Studies

#### 4.4.1. Long-Term Storage of the Dispersion

The long-term colloidal stability of the HNP-siHOXB7 kept at 4 °C was evaluated at different time points after preparation (0, 15, 30, 90, and 180 days) by mean hydrodynamic diameter and polydispersity index, as described in Section 4.3.

#### 4.4.2. Lyophilization

Firstly, HNP-siHOXB7 was either mixed or not mixed with cryoprotectant sucrose 10% (*w*/*v*), frozen with liquid nitrogen, and submitted to the lyophilization process using LD Plus Alpha 1–2 (Martin Christ, Osterode am Harz, Germany) equipment at 0.064 mbar and −53 °C, for 24 h. The resulting powder was resuspended in Milli-Q water, and the mean hydrodynamic diameter and polydispersity index were evaluated before and after the procedure, as well as after 10 days of resuspension.

### 4.5. Encapsulation Efficiency

The encapsulation efficiency of HOXB7 siRNA into NP was indirectly determined through the quantification of non-encapsulated siRNA, as described by Pittella et al. [21]. The methodology is similar to the purification process described in topic 4.2. After centrifugation, the non-encapsulated siRNA filtrate was collected and analyzed in Nanodrop Spectrophotometer ND 1000 (ThermoScientific, Waltham, MA, USA) at 260 nm. The percentage of loaded siRNA was calculated using the following formula:

Encapsulated percentage (%) = (total siRNA content − free siRNA/total siRNA content) × 100(1)

### 4.6. In Vitro Assays

The Luminal A subtype of human breast cancer cell line, MCF-7 (ATCC number: HTB-22), was used in every assay and cultured in RPMI medium supplemented with 10% FBS and 1% antibiotics. The cells were maintained in incubation at 37 °C and 5% CO_2_.

#### 4.6.1. Cell Viability Assay

Cell viability was evaluated by MTT [3-(4, 5-dimethylthiazol-2-yl)-2, 5-diphenyltetrazolium bromide] assay. Cells were incubated for adherence overnight in the same conditions presented before after seeding (5000 cells/well) in a 96-well plate. Treatment (HNP-siHOXB7) and controls (HNP-mock, free siHOXB7, and medium) were added and replaced by MTT after 24 h and 48 h of incubation. After 4 h of MTT incubation, formazan crystals were solubilized with DMSO. The absorbance was then measured at 540 nm in a microplate reader.

#### 4.6.2. Scratch Assay

A scratch test was performed in monolayered MCF-7 cells (50,000 cells/well in a 24-well plate) with the help of a sterile 200 µL tip. Cells were washed with PBS 1× for removal of unattached cells and treatment (HNP-siHOXB7), and controls (full medium and free siHOXB7) were added to different wells. Images were obtained and evaluated using an inverted optical microscope Leica DMI6000B coupled to a Hamamatsu Orca-ER com camera at 40× magnification at different time points (0 h, 6 h, 24 h, and 48 h). The acquired images were treated and processed using ImageJ software, version 1.52a (NIH, Bethesda, MD, USA), and the free area was determined.

### 4.7. Hemolytic Activity

The hemolytic activity was evaluated according to Silva et al. [38]. Fresh erythrocyte samples were collected and separated by centrifugation at 2000 rpm for 5 min. The samples were washed twice with saline solution and resuspended in 10 mL (erythrocytes corresponding to 1 mL of blood). Aliquots of NP-siHOXB7 (10, 50, 100, 150, and 200 nM) and NP-mock (200 nM) were added to erythrocyte suspensions (10%:90%). The negative and positive controls were prepared by adding saline solution and 0.1% Triton X-100 solution, respectively. Afterward, the samples were incubated under agitation for 1 h at 37 °C and later centrifuged at 2000 rpm for 5 min. Visual analysis was performed, as well as UV/Vis spectrometry at 415 nm of supernatants and microscopic images. The hemolysis rate (%) was determined by the following formula:

Hemolysis rate (%) = (Abs sample − Abs negative/Abs positive) × 100(2)

### 4.8. In Vivo Assay

Animal models of mice bearing human breast tumors were prepared according to Zhang et al. [39], with slight modifications. Briefly, immunosuppressed female Nude mice (6–8 weeks old) were injected subcutaneously in the right lower abdominal breast with 2 million MCF-7 cells after a 1 week of estrogen pre-treatment (1.5 mg/kg, i.p.) since this cell line is estrogen dependent. Breast tumors were allowed to grow for 20 and 30 days in early-stage and advanced-stage groups, and the animals in each group were randomly distributed into three treatment groups.

#### 4.8.1. Antitumoral Activity

Antitumoral effect on early-stage tumors (50 mm^3^) was evaluated in three groups: TMX (n = 4), HNP-siHOXB7 + TMX (n = 4) and saline control (n = 4). On the other hand, advanced tumors (250 mm^3^) were treated with HNP-siHOXB7 (n = 5), HNP-siHOXB7 + TMX (n = 4), and saline control (n = 4). HNP-siHOXB7 treatment was administered by intravenous injection in the tail vein (25 µg of siRNA) on days 1, 4, 8, 10, and 13. TMX (10 mg/kg) was intraperitoneally administered every 2 days, as well as estrogen. The dose regimens were based on previous studies [37,40]. Tumor size and body weight were monitored constantly until day 14, in which animals were euthanized. Blood samples, tumor tissue, and other organs (heart, lung, kidney, liver, and spleen) were collected for further investigations. Tumor volume was calculated based on the following modified ellipsoidal formula:

Volume = 1/2 (length × width^2^) (3)

#### 4.8.2. HOXB7 Gene Expression Evaluation

Real-time PCR (qPCR) was performed to evaluate HOXB7 mRNA levels after 24 h of HNP-siHOXB7 last administration. Pi-Clear RNA Total (Pi-Biotech, Juiz de Fora, Brazil) was used for total RNA extraction from tumor tissue (100 mg) as described by the manufacturer. After cDNA synthesis using a High-Capacity cDNA Reverse Transcription kit (Applied Biosystems, Waltham, MA, USA), qPCR was carried out using qPCR-SYBR-Green mix (Ludwig Biotecnologia Ltd.a, Alvorada, Brazil) and specific primers as described in Section 4.1. and using a 7500 Fast Real-time PCR instrument (Applied Biosystems, Waltham, MA, USA). The temperature cycle was performed as follows: (1) holding stage was performed at 50 °C for 2 min and 95 °C for 2 min, followed by (2) 60 cycles at 95 °C for 15 s and 60 °C for 1 min. HOXB7 gene expression was normalized by human β-actin as a house-keeper gene.

#### 4.8.3. Histopathological Analysis

The collected organs and tumors were fixed with 4% formaldehyde for 24 h, further dehydrated in alcohol solutions, and finally embedded in paraffin, according to [31]. The paraffin blocks were sectioned (5 μm) and stained with hematoxylin and eosin. The histological slides were analyzed, and representative images were captured.

#### 4.8.4. Hematological and Biochemical Parameters Analysis

General toxicity in mice was evaluated 24 h after the last injection through blood analysis. Whole blood was collected by heart puncture and transferred to microtubes containing heparin. Hematological and biochemical analysis were carried out as routine blood analysis in the Laboratório Veterinário Vivanálises (Juiz de Fora, Brazil). The total and differential white blood cell (WBC), red blood cells (RBC), hemoglobin concentration (HGB), hematocrit percentage (HCT), mean corpuscular volume (MCV), mean corpuscular hemoglobin concentration (MCHC) and platelets (PLT) were measured, as well as, plasma protein, aspartate aminotransferase (AST), alanine transaminase (ALT) urea and creatinine.

### 4.9. Statistical Analysis

Analysis of variance (ANOVA) followed by Turkey post hoc test was performed to evaluate the treatment effects and compare individual groups, respectively. GraphPad Prism 5.0 software (GraphPad Software, Inc., La Jolla, CA, USA) was used for statistical analysis, and significative differences were considered as *p* < 0.05. The results were expressed as mean values ± standard error mean (SEM).

## 5. Conclusions

In this study, a non-toxic PEG-pGlu calcium phosphate nanocarrier for HOXB7 siRNA was evaluated both in vitro and in animal models bearing Luminal A breast tumors and showed promising capacity for gene delivery without presenting cytotoxicity. HOXB7 silencing was effective in controlling tumoral growth with and without TMX combination. Furthermore, our results support that HOXB7 silencing associated with TMX promoted a better prognosis and extended general survival without severe hepatological and renal toxicity in mice. Nevertheless, optimization of HOXB7 siRNA concentration may be interesting for enhancing the RNAi effect and re-sensitize tumor cells to TMX. This approach may even lead to the reduction in TMX doses while decreasing the adverse effects of this chemotherapeutic agent. Finally, a longer treatment period must be considered due to the intrinsic characteristics of the specific molecular subtype of the tumor to improve the RNAi antitumoral effect and synergic effect with TMX.

## Figures and Tables

**Table 1:** Mean hydrodynamic diameter, polydispersity index (PDI), and Zeta potential of hybrid nanoparticles.

	Hydrodynamic Diameter (nm)	PDI	Zeta Potential (mV)
HNP-siHOXB7	74.4 ± 5.5	0.1 ± 0.03	−1.16 ± 0.95
HNP-mock	82.2 ± 14.5	0.1 ± 0.02	−0.86 ± 0.1

Results are expressed as mean ± standard error of the mean (n = 3).

**Table 2:** Mean hydrodynamic diameter and polydispersity index (PDI) of hybrid nanoparticles before and after the purification process.

		Hydrodynamic Diameter (nm)			PDI			
	Before	After	6 d.a.	17 d.a.	Before	After	6 d.a.	17 d.a.
HNP-mock	79.83 ± 0.03	78.38 ± 0.2	82.57 ± 0.33	88.44 ± 0.46 *	0.08 ± 0	0.08 ± 0	0.08 ± 0	0.07 ± 0
HNP-siHOXB7	70.78 ± 0.03	68.08 ± 044	71.4 ± 0.25	73.66 ± 0.27 *	0.07 ± 0	0.06 ± 0	0.06 ± 0	0.06 ± 0

d.a.: days after purification. Results are expressed as mean ± standard error of the mean (n = 3). ANOVA followed by Bonferroni (* *p* < 0.05).

**Table 3:** Stability of hybrid nanoparticles before and after (1 day and 10 days) freeze-drying process with and without cryopreservant.

	Hydrodynamic Diameter (nm)			PDI		
	Before	After	10 d.a.	Before	After.	10 d.a.
HNP	68.51 ± 0.2	74.33 ± 1.2	80.1 ± 0.55	0.03 ± 0	0.15 ± 0.03	0.17 ±0.02
HNP + Sucrose 10%	81.21 ± 0.1	79.4 ± 0.4	151.6 ± 7 *	0.18 ± 0	0.19 ± 0	0.66 ± 0.07

d.a.: days after freeze drying process. Results are expressed as mean ± standard error of the mean (n = 3). ANOVA followed by Bonferroni (* *p* < 0.05).

**Table 4:** Hematological and biochemical parameters after cotreatment and free TMX in early stages tumor animals.

**Hematological Parameters**							
**Parameters**	**Control**	**HNP + TMX**	**TMX**	**Parameters**	**Control**	**HNP + TMX**	**TMX**
RBC (×10^6^/mm^3^)	7.9 ± 0.2	7.82 ± 0.2	7.5 ± 0.1	Banded neutrophils (mm^3^)	0	0	0
HGB (g/dL)	12.05 ± 0.2	12.23 ± 0.4	11.53 ± 0.1	Segmented neutrophils (mm^3^)	2582 ± 101.4	1343 ± 182.3 *	1184 ± 274 *
HCT (%)	46 ± 1	46.25 ± 0.9	43 ± 0.4	Eosinophil (mm^3^)	16 ± 10.8	8.5 ± 5.5	9 ± 9
MCV (fL)	57.75 ± 0.3	59.13 ± 0.4	57.26 ± 1.1	Lymphocyte (mm^3^)	284.3 ± 178.2	224.3 ± 101.8	246 ± 5
MCHC (g/dL)	26.2 ± 0.2	26.41 ± 0.3	26.81 ± 0.4	Monocyte (mm^3^)	284.5 ± 93.5	144 ± 38.2	161 ± 3
WBC (mm^3^)	3100 ± 601.4	2300 ± 288.7	1175 ± 265.8 *	Basophil (mm^3^)	0	0	0
Metamyelocyte (mm^3^)	0	0	0	PLT (mil/mm^3^)	472.3 ± 23.2	558.3 ± 57.2	612.5 ± 51.1
**Biochemical Parameters**							
**Parameters**	**Control**	**HNP + TMX**	**TMX**	**Parameters**	**Control**	**HNP + TMX**	**TMX**
Urea (mg/dL)	47.51 ± 7.6	41.3 ± 3.5	46.78 ± 2.9	ALT (U/L)	60.4 ± 2.8	55.65 ± 8.6	40.35 ± 0.6
Creatinine (mg/dL)	0.4 ± 0.02	0.57 ± 0.04 *	0.42 ± 0.03	AST (U/L)	218 ± 28.6	175.5 ± 13.3	170.5 ± 4.2
Plasma protein	6.1 ± 0.1	5.65 ± 0.1 *	5.3 ± 0.1 *				

Results are expressed as mean ± standard error of the mean (SEM). ANOVA followed by Bonferroni (* *p* < 0.05). RBC: red blood cells; HGB: hemoglobin concentration; HCT: hematocrit percentage; MCV: mean corpuscular volume; MCHC: mean corpuscular hemoglobin concentration; WBC: white blood cells; PLT: platelets; ALT: alanine aminotransferase; AST: aspartate aminotransferase.

## Data Availability

Data are contained within the article and Supplementary Materials.

## Supplementary Materials

The following supporting information can be downloaded at https://www.mdpi.com/article/10.3390/ph17101325/s1. Figure S1: Colloidal stability of non-purified HNP-siHOXB7 (square, n = 3) and HNP-mock (circle, n = 3) stored at 4 °C for 180 days. Mean hydrodynamic diameter (black) and PDI (red). Results are expressed as mean ± standard error of the mean. ANOVA followed by Bonferroni (* *p* < 0.05). Table S1: Mean hydrodynamic diameter and polydispersity index (PDI) of hybrid nanoparticles before and after 180 days of storage at 4 °C.

## References

[1] Bray F., Ferlay J., Soerjomataram I., Siegel R.L., Torre L.A., Jemal A. (2018). Global cancer statistics 2018: GLOBOCAN estimates of incidence and mortality worldwide for 36 cancers in 185 countries. CA Cancer J. Clin..

[2] Sonkin D., Thomas A., Teicher B.A. (2024). Cancer treatments: Past, present, and future. Cancer Genet..

[3] Nascimento R.G., Otoni K.M. (2020). Histological and molecular classification of breast cancer: What do we know?. Mastology.

[4] Hoefnagel L.D., Moelans C.B., Meijer S.L., van Slooten H.J., Wesseling P., Wesseling J., Westenend P.J., Bart J., Seldenrijk C.A., Nagtegaal I.D. (2012). Prognostic value of estrogen receptor α and progesterone receptor conversion in distant breast cancer metastases. Cancer.

[5] Jin K., Kong X., Shah T., Penet M.F., Wildes F., Sgroi D.C., Ma X.J., Huang Y., Kallioniemi A., Landberg G. (2012). The HOXB7 protein renders breast cancer cells resistant to tamoxifen through activation of the EGFR pathway. Proc. Natl. Acad. Sci. USA.

[6] Caré A., Silvani A., Meccia E., Mattia G., Peschle C., Colombo M.P. (1998). Transduction of the SkBr3 breast cancer carcinoma cell line with the HOXB7 gene induces bFGF expression, increases cell proliferation, and reduces growth factor dependence. Oncogene.

[7] Wu X., Chen H., Parker B., Rubin E., Zhu T., Lee J.S., Argani P., Sukumar S. (2006). HOXB7, a homeodomain protein, is overexpressed in breast cancer and confers epithelial-mesenchymal transition. Cancer Res..

[8] Rubin E., Wu X., Zhu T., Cheung J.C., Chen H., Lorincz A., Pandita R.K., Sharma G.G., Ha H.C., Gasson J. (2007). A role for the HOXB7 homeodomain protein in DNA repair. Cancer Res..

[9] Jin K., Park S., Teo W.W., Korangath P., Cho S.S., Yoshida T., Győrffy B., Goswami C.P., Nakshatri H., Cruz L.A. (2015). HOXB7 is an ERα cofactor in the activation of HER2 and multiple ER target genes leading to endocrine resistance. Cancer Discov..

[10] Jin K., Sukumar S. (2015). A pivotal role for HOXB7 protein in endocrine-resistant breast cancer. Oncoscience.

[11] Urits I., Swanson D., Swett M.C., Patel A., Berardino K., Amgalan A., Berger A.A., Kassem H., Kaye A.D., Viswanath O. (2020). A Review of Patisiran (ONPATTRO^®^) for the Treatment of Polyneuropathy in People with Hereditary Transthyretin Amyloidosis. Neurol. Ther..

[12] Menck C.F.M. (2010). A nova grande promessa da inovação em fármacos: RNA interferência saindo do laboratório para a clínica. Estud. Avançados.

[13] Mishra A., Srivastava A., Pateriya A., Tomar M.S., Mishra A.K., Shrivastava A. (2021). Metabolic reprograming confers tamoxifen resistance in breast cancer. Chem.-Biol. Interact..

[14] Aleixo D.T., Gualberto A.C.M., Valle A.B.C.S., Da Silva L.C., Ferreira K.C.B., Lemos A.S.O., Fabri R.L., Tavares G.D., Cazarim M.S., Gameiro J. (2024). Macauba oil carried by polymeric micelles reduces migration and proliferation of triple-negative breast cancer cells. RSC Pharm..

[15] Ferreira K.C.B., Valle A.B.C.S., Gualberto A.C.M., Aleixo D.T., Silva L.M., Santos M.M., Costa D.S., Oliveira L.L., Gameiro J., Tavares G.D. (2022). Kaurenoic acid nanocarriers regulates cytokine production and inhibit breast cancer cell migration. J. Control. Release.

[16] Rezaei S., Kashanian S., Bahrami Y., Cruz L.J., Motiei M. (2020). Redox-Sensitive and Hyaluronic Acid-Functionalized Nanoparticles for Improving Breast Cancer Treatment by Cytoplasmic 17α-Methyltestosterone Delivery. Molecules.

[17] Gomhor J., Alqaraghuli H., Kashanian S., Rafipour R., Mahdavian E., Mansouri K. (2018). Development and characterization of folic acid-functionalized apoferritin as a delivery vehicle for epirubicin against MCF-7 breast cancer cells. Artif. Cells Nanomed. Biotechnol..

[18] Moazzam M., Zhang M., Hussain A., Yu X., Huang J., Huang Y. (2024). The landscape of nanoparticle-based siRNA delivery and therapeutic development. Mol. Ther..

[19] Whitehead K., Langer R., Anderson D. (2009). Knocking down barriers: Advances in siRNA delivery. Nat. Rev..

[20] Kakizawa Y., Kataoka K. (2002). Block copolymer self-assembly into monodispersive nanoparticles with hybrid core of antisense DNA and calcium phosphate. Langmuir.

[21] Pittella F., Zhang M., Lee Y., Kim H.J., Tockary T., Osada K., Ishii T., Miyata K., Nishiyama N., Kataoka K. (2011). Enhanced endosomal escape of siRNA-incorporating hybrid nanoparticles from calcium phosphate and PEG-block charge conversional polymer for efficient gene knockdown with negligible cytotoxicity. Biomaterials.

[22] de Mello L.J., Souza G.R.R., Winter E., Silva A.H., Pittella F., Creczynski-Pasa T.B. (2017). Knockdown of antiapoptotic genes in breast cancer cells by siRNA loaded into hybrid nanoparticles. Nanotechnology.

[23] Souza G.R.R., Dalmina M., Restrepo J.A.S., de Mello Junior L.J., Silva A.H., Gualberto A., Gameiro J., Dittz D., Pasa A.A., Pittella F. (2021). Short interfering RNA delivered by a hybrid nanoparticle targeting VEGF: Biodistribution and anti-tumor effect. Biochim. Biophys. Acta Gen. Subj..

[24] Gao P., Zhang X., Wang H., Zhang Q., Li H., Li Y., Duan Y. (2016). Biocompatible and colloidally stabilized mPEG-PE/calcium phosphate hybrid nanoparticles loaded with siRNAs targeting tumors. Oncotarget.

[25] Nangia S., Sureshkumar R. (2012). Effects of nanoparticle charge and shape anisotropy on translocation through cell membranes. Langmuir.

[26] Matsumara Y., Maeda H. (1986). A new concept for macromolecular therapeutics in cancer chemotherapy: Mechanism of tumoritropic accumulation of proteins and the antitumor agent smancs. Cancer Res..

[27] Kakizawa Y., Furukawa S., Kataoka K. (2004). Block copolymer-coated calcium phosphate nanoparticles sensing intracellular environment for oligodeoxynucleotide and siRNA delivery. J. Control. Release.

[28] Perrault S.D., Walkey C., Jennings T., Fischer H.C., Chan W.C. (2009). Mediating tumor targeting efficiency of nanoparticles through design. Nano Lett..

[29] Statefeld J., Mckenna S., Patel T. (2016). Dynamic light scattering: A practical guide and applications in biomedical sciences. Biophys. Ver..

[30] Winkler A.W., Hoff H.E., Smith P.K. (1940). Cardiovascular effects of potassium, calcium, magnesium, and barium: An experimental study of toxicity and rationale of use in therapeutics. Yale J. Biol. Med..

[31] Zhou Q., Wang Y., Xiang J., Piao Y., Zhou Z., Tang J., Liu X., Shen Y. (2018). Stabilized calcium phosphate hybrid nanocomposite using a benzoxaborole-containing polymer for pH-responsive siRNA delivery. Biomater. Sci..

[32] Hosonuma M., Yoshimura K. (2023). Association between pH regulation of the tumor microenvironment and immunological state. Front. Oncol..

[33] Valle A.B.C.S., Gualberto A.C., Ferreira K.C.B., Creczynski-Pasa T.B., Gameiro J., Tavares G.D., Pittella F. (2021). HOXB7 siRNA Delivered by Hybrid Nanoparticles and the Co-Therapy with Tamoxifen: Promising Strategy against Hormone Receptor-Positive Breast Cancer. Mater. Proc..

[34] Thapa R.K., Kim J.O. (2023). Nanomedicine-based commercial formulations: Current developments and future prospects. J. Pharm. Investig..

[35] Manaargadoo-Catin M., Ali-Cherif A., Pougnas J.L., Perrin C. (2015). Hemolysis by surfactants—A review. Adv. Colloid Interface Sci..

[36] Liu H., Dong A., Rasteh A.M., Wang P., Weng J. (2024). Identification of the novel exhausted T cell CD8+ markers in breast cancer. Sci. Rep..

[37] Pittella F., Miyata K., Maeda Y., Suma T., Watanabe S., Chen Q., Christie R.J., Osada K., Nishiyama N., Kataoka K. (2012). Pancreatic cancer therapy by systemic administration of VEGF siRNA contained in calcium phosphate/charge-conversional polymer hybrid nanoparticles. J. Control. Release.

[38] Silva L.M., Marconato D.G., Nascimento da Silva M.P., Barbosa Raposo N.R., Faria Silva Facchini G., Macedo G.C., Teixeira F.S., Barbosa da Silveira Salvadori M.C., Faria Pinto P., Moraes J. (2021). Licochalcone A-loaded solid lipid nanoparticles improve antischistosomal activity in vitro and in vivo. Nanomedicine.

[39] Zhang Y., Leonard M., Shu Y., Yang Y., Shu D., Guo P., Zhang X. (2016). Overcoming Tamoxifen Resistance of Human Breast Cancer by Targeted Gene Silencing Using Multifunctional pRNA Nanoparticles. ACS Nano.

[40] Raha P., Thomas S., Thurn K.T., Park J., Munster P.N. (2015). Combined histone deacetylase inhibition and tamoxifen induces apoptosis in tamoxifen-resistant breast cancer models, by reversing Bcl-2 overexpression. Breast Cancer Res..

