# Supplementary material for: In Vivo HOXB7 Gene Silencing and Cotreatment with Tamoxifen for Luminal A Breast Cancer Therapy
Source: Pharmaceuticals (Basel). 2024 Oct 4;17(10):1325. doi: 10.3390/ph17101325 (PMC11509954; doi:10.3390/ph17101325)
Supplement: Supplementary file 1 [file pharmaceuticals-17-01325-s001.zip › pharmaceuticals-3229787-supplementary.pdf]

SUPPLEMENTARY MATERIAL

In vivo HOXB7 gene silencing and cotreatment with tamoxifen for Luminal A breast cancer therapy

Ana Beatriz Caribé dos Santos Valle[1], Fábio Fernando Alves da Silva[2], Maria Ângela Pepe Carneiro[2], Bruno Espuche[3], Guilherme Diniz Tavares[1], Emerson Soares Bernardes[2], Sergio Enrique Moya[3], Frederico Pittella[1]

[1] Laboratório de Desenvolvimento de Sistemas Nanoestruturados, Faculdade de Farmácia, Universidade Federal de Juiz de Fora, Rua José Lourenço Kelmer, 36036-900, Juiz de Fora, MG, Brasil; abcsvalle@hotmail.com; guilherme.tavares@ufjf.br; frederico.pittella@ufjf.br
[2] Instituto de Pesquisas Energéticas e Nucleares, Centro de Radiofarmácia (IPEN/CECRF), Comissão Nacional de Energia Nuclear, São Paulo 05508-000, Brazil; fabiofufg@gmail.com, mangelapc@usp.br, emerson.bernardes@gmail.com
[3] Soft Matter Laboratory, Centro de Investigación Cooperativa en Biomateriales, Paseo Miramón, 182, 20014, San Sebastián, Spain; smoya@cicbiomagune.es

RESULTS

Colloidal stability studies

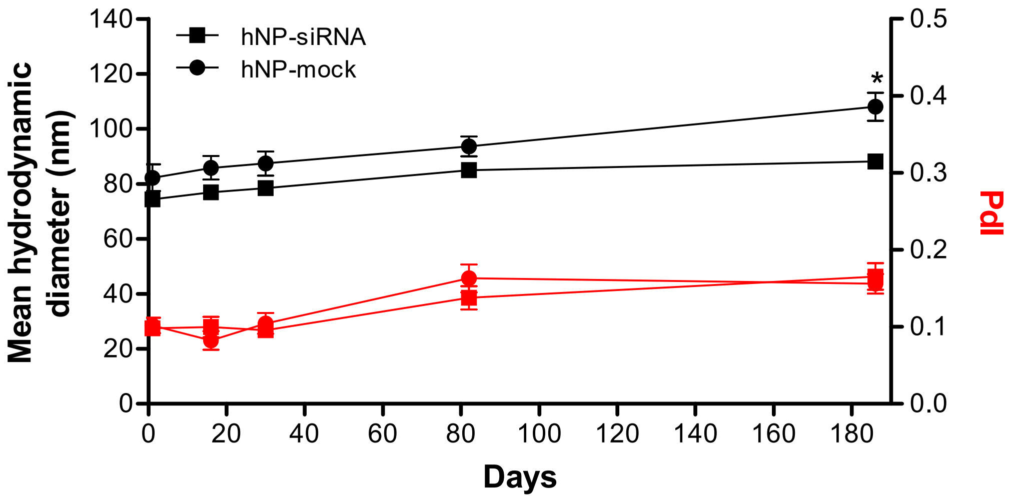

Figure S1. Colloidal stability of non-purified HNP-siHOXB7 (square, n = 3) and HNP-mock (circle, n = 3) stored at 4 °C for 180 days. Mean hydrodynamic diameter (black) and PdI (red). Results are expressed as mean ± standard error of the mean. ANOVA followed by Bonferroni (*p<0.05).

Table S1. Mean hydrodynamic diameter and polydispersity index (PdI) of hybrid nanoparticles before and after 180 days storage at 4 °C.

	**Hydrodynamic diameter (nm)**		**PdI**	
	Day 1	Day 180	Day 1	Day 180
**HNP-siHOXB7**	74.4 ± 1.8	88.16 ± 1.9	0.1 ± 0.01	0.15 ± 0.01
**HNP-mock**	82.2 ± 4.8	108 ± 1.9 *	0.1 ± 0	0.16 ± 0.01

Results are expressed as mean ± standard error of the mean (n = 3). ANOVA followed by Bonferroni (*p<0.05).
